# Wavelength-Scanning SPR Imaging Sensors Based on an Acousto-Optic Tunable Filter and a White Light Laser

**DOI:** 10.3390/s17010090

**Published:** 2017-01-05

**Authors:** Youjun Zeng, Lei Wang, Shu-Yuen Wu, Jianan He, Junle Qu, Xuejin Li, Ho-Pui Ho, Dayong Gu, Bruce Zhi Gao, Yonghong Shao

**Affiliations:** 1 College of Optoelectronic Engineering, Key Laboratory of Optoelectronic Devices and Systems of Ministry of Education and Guangdong Province, Shenzhen Key Laboratory of Sensor Technology, Shenzhen University, Shenzhen 518060, China; zyoujun@yeah.net (Y.Z.); wanglei_mn@yahoo.com (L.W.); jlqu@szu.edu.cn (J.Q.); lixuejin@szu.edu.cn (X.L.); 2 Department of Electronic Engineering, The Chinese University of Hong Kong, Shatin 999077, Hong Kong, China; sywu@ee.cuhk.edu.hk (S.-Y.W.); hpho@ee.cuhk.edu.hk (H.-P.H.); 3 Shenzhen Entry-exit Inspection and Quarantine Bureau, Shenzhen 518033, China; hejianan6398@163.com (J.H); wanhood@163.com (D.G.); 4 Department of Bioengineering and COMSET, Clemson University, Clemson, SC 29634, USA; zgao@clemson.edu

**Keywords:** surface plasmon resonance, SPR imaging, microarray analysis, wavelength scanning, acousto-optic tunable filter (AOTF), white light laser

## Abstract

A fast surface plasmon resonance (SPR) imaging biosensor system based on wavelength interrogation using an acousto-optic tunable filter (AOTF) and a white light laser is presented. The system combines the merits of a wide-dynamic detection range and high sensitivity offered by the spectral approach with multiplexed high-throughput data collection and a two-dimensional (2D) biosensor array. The key feature is the use of AOTF to realize wavelength scan from a white laser source and thus to achieve fast tracking of the SPR dip movement caused by target molecules binding to the sensor surface. Experimental results show that the system is capable of completing a SPR dip measurement within 0.35 s. To the best of our knowledge, this is the fastest time ever reported in the literature for imaging spectral interrogation. Based on a spectral window with a width of approximately 100 nm, a dynamic detection range and resolution of 4.63 × 10^−2^ refractive index unit (RIU) and 1.27 × 10^−6^ RIU achieved in a 2D-array sensor is reported here. The spectral SPR imaging sensor scheme has the capability of performing fast high-throughput detection of biomolecular interactions from 2D sensor arrays. The design has no mechanical moving parts, thus making the scheme completely solid-state.

## 1. Introduction

The surface plasmon resonance (SPR) sensing technique, which offers unique real-time and label-free measurement capabilities with high detection sensitivity, has become an important tool for exploring the kinetics of biomolecular interactions and has been widely used to detect chemical and biological analytes [1,2,3,4]. By combining the SPR approach with an imaging system, high-throughput, real-time, label-free biosensing in 2D microarrays and parallel monitoring of a multiple number of biomolecular interactions can be achieved [5,6,7,8,9]. However, to become a potential candidate to replace the well-established fluorescence-based microarray technique, which suffers from photobleaching and the lack of quantitative information, the data capture speed and detection sensitivity of the SPR imaging approach need to be further improved. 

The SPR phenomenon produces a minimum in the reflectivity (SPR dip) when the incident p-polarized light resonantly couples with a surface plasma wave (SPW) at a specific angle or wavelength. The resonant coupling occurs when:

(1) $$k_{x}=\frac{2{\pi n}_{p}}{\lambda }\sin\theta \approx \frac{2\pi }{\lambda }\sqrt{\frac{\varepsilon_{\mathrm{mr}}n_{s}^{2}}{\varepsilon_{\mathrm{mr}}+n_{s}^{2}}}=k_{\mathrm{sp}}$$

where **k_x_** is the tangential wave vector of the p-polarized incident electromagnetic field, **k_sp_** is the SPW vector, n_p_ is the refractive index of the prism, *λ* is the wavelength of excitation light, $\varepsilon_{\mathrm{mr}}$ is the real part of a complex dielectric constant of Au, θ is the angle of incidence, and n_s_ is the refractive index of the sample at the metal/dielectric interface [10,11].

The position of the SPR dip along the spectrum is highly sensitive to changes in the refractive index of the sample at the metal/dielectric interface. Analyte binding onto the sensor surface will induce a change of the refractive index at the metal/dielectric interface, thus making it possible to detect interactions among biological molecules in the vicinity of the metal/dielectric interface by tracking the location of the SPR dip. 

The four main types of SPR sensing techniques are angle modulation, spectral modulation, intensity modulation, and phase modulation [12]. To perform real-time high-throughput SPR sensing measurements, intensity modulation SPR systems are typically adopted. In intensity modulation mode, both the incident wavelength and the incident angle are fixed to a value close to the optimum sensitivity for the binding interactions monitored. In practice, some significant data dispersion and non-uniform response on the entire sensing surface limit the use of the intensity modulation SPR sensors [6]. Although phase modulation SPR sensing has the highest sensitivity among these SPR sensing techniques, the inherent small dynamic detection range limits their use. The angle modulation and spectral modulation techniques have been widely studied because they can overcome these limitations [6,13,14]. 

In angle modulation mode, the incident wavelength is fixed, the angle-intensity curve is obtained by scanning the angle of incidence. Then, the corresponding resonance angle of the sample is obtained through data analysis. Several methods have been reported to improve scanning speed, including a converging excitation beam [15,16] or diverging excitation beam [17,18,19] and a 1D acousto-optical deflector (AOD) scanner [20] or controlled mirror scanning [13].

Spectral modulation SPR can achieve sensitivity and dynamic detection range similar to angle modulation SPR. Moreover, spectral modulation SPR is much more flexible for optimization because a range of operation wavelengths can be freely selected to obtain the best SPR excitation [6]. In spectral modulation mode, a broadband light is usually used, and the SPR spectral profile of the sample can be obtained either by scanning the incidence wavelength or by using a spectrometer for analyzing the reflected beam [21,22,23]. The sample’s corresponding resonant wavelength can be obtained by data analysis. Spectrometer-based SPR sensing techniques can achieve fast detection of single sensor site or even one-dimensional line arrays [24,25]. However, this is not a straightforward method for detecting two-dimensional (2-D) arrays. Yuk et al. achieved 2D imaging of a 2 mm pot in about 180 s by using a fiber optic spectrometer [26]. Liu et al. reported a 1D optical line scan spectral SPR system for imaging 2D arrays. For an area of 8 mm × 8 mm, it took 60 s to measure the SPR dips of the 2D array [27]. Wong et al. developed a scanless 2D spectral SPR imaging sensor based on the combination of a polarization control scheme and a color CCD camera. It realized 2D spectral SPR imaging with high sensitivity (2.7 × 10^−6^ RIU) in a linear response range from 1.3333–1.3365 RIU [28]. Recently, to improve the speed of measurement, a specially fitted algorithm based on using five parameters has been developed for the spectral SPR imaging sensors [29]. The measurement time of the SPR dip has been shortened dramatically, to 10 s in 2D arrays. More recently, fast 2D spectral SPR imaging based on a liquid crystal tunable filter (LCTF) has been reporgted [30,31,32].

Here, we describe a fast spectral SPR imaging system (SPRi) based on an AOTF scanning of the incident wavelength. AOTF scan of a white light laser provides a flexible wavelength-scanning excitation light source. With an SPR dip tracking algorithm, the time for obtaining an SPR dip is about 0.35 s.

## 2. Experimental Section

### 2.1. Algorithm

**Figure 1:**
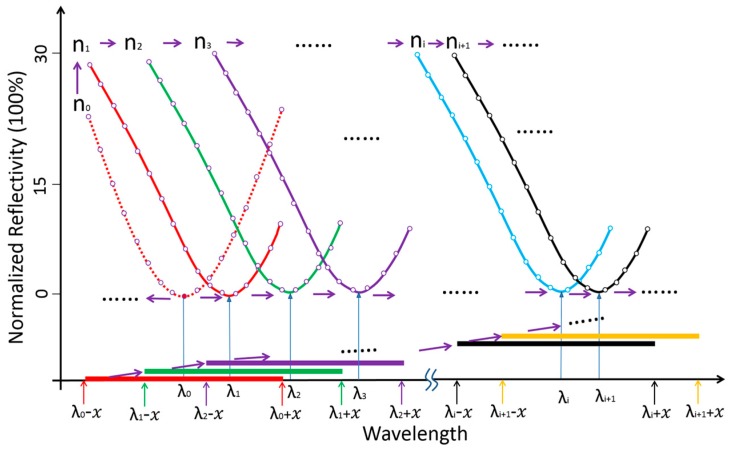
Schematic of tracking the SPR dip based on the feedback loop technique. Here *n*_0_, *n*_1_, …, *n_i_* and *n_i_*_+1_ represent a series of refractive index values at the Au/sample interface when a molecular binding reaction occurs on the sensor surface. As the refractive index changes continuously from *n*_0_ to *n_i_*, the spectral range to be scanned is guided by the feedback loop and automatically adjusted from the original spectral range [$\lambda_{0}-x$, $\lambda_{0}+x$] to [$\lambda_{i}-x$, $\lambda_{i}+x$]. This way, only a selected range around the resonance dip will be interrogated at any time, thereby drastically shortening the time necessary to calculate the location of the SPR dip.

Figure 1 demonstrates operation of the feedback loop. An initial SPR spectral profile is obtained by scanning the incident wavelength across a large spectral range to extract the baseline resonant wavelength $\lambda_{0}$. Then, a spectral range from $\lambda_{0}-x$ to $\lambda_{0}+x$, with which one can obtain the desired SPR dip with sufficient resolution (red solid straight line along horizontal axis) is chosen. The corresponding partial SPR plot near the SPR dip (red dotted curve) will be repeated if the refractive index *n*_0_ of the baseline solution does not change.

When a change of refractive index occurs, e.g., from *n*_0_ to *n*_1_, a new partial SPR plot (red solid curve) is obtained by scanning the incident wavelength from $\lambda_{0}-x$ to $\lambda_{0}+x$. Thus a new resonant wavelength $\lambda_{1}$ will be extracted; then the new scanning spectral range will be modified to $\lambda_{1}-x$ to $\lambda_{1}+x$ (green solid straight line along the horizontal axis). If the refractive index continues to change from *n*_1_ to *n*_2_, the corresponding partial SPR plot (green solid curve) is also obtained by scanning the incident wavelength from $\lambda_{1}-x$ to $\lambda_{1}+x$. This process continues until the system has identified the next new resonant wavelength $\lambda_{2}$, which triggers further modification of the scanning range, i.e., from $\lambda_{2}-x$ to $\lambda_{2}+x$. In a more general representation, the (*i +* 1)th scanning spectral range, which covers $\lambda_{i+1}-x$ to $\lambda_{i+1}+x$ (yellow solid straight line along horizontal axis), is generated from the resonant wavelength extracted from the partial SPR plot near the SPR dip obtained in the *i*th scanning spectral range of $\lambda_{i}-x$ to $\lambda_{i}+x$ (black solid straight line along horizontal axis). For a 2D sensor array, a number of different resonant wavelengths are obtained from the sensor elements because each element has been designated to perform a specific sensing task. In such a situation, our system will try to cover the entire range of all the elements by registering the upper and lower limits, i.e., $\lambda_{i\min}$ and $\lambda_{i\max}$, of the spectral dip, and the range can be calculated using the following expressions:

(2) $$\lambda_{\mathrm{begin}}=\lambda_{i\min}-x$$

(3) $$\lambda_{\mathrm{end}}=\lambda_{i\max}+x$$

in which *λ*_begin_ and *λ*_end_ are respectively the first and last wavelengths of the *i*th scanning spectral range. 

### 2.2. Setup

**Figure 2:**
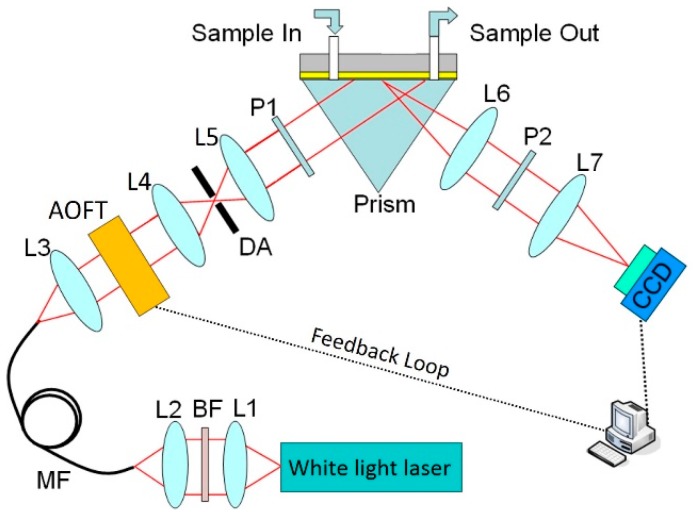
Schematic of our SPRi system in the Kreschmann configuration. Light from a white light laser is collected by a multimodal fiber through a set of coupling optics. At the exit end of the fiber, the beam is collimated by a group of lenses and spatially filtered by an aperture before passing through the tunable spectral filter unit and a linear polarizer. The sensor surface is imaged by a CCD camera using two imaging lenses. L1–L7: lens; BF: bandpass filter; MF: multimode fiber; DA: diaphragm aperture; AOTF: acousto-optic tunable filter; P1 and P2: polarizer.

Figure 2 shows a schematic of our experimental setup. White light laser (SC-PRO, YSL, Wuhan, China) is generated through ultrashort pulse laser pumping photonic crystal fiber. Compared to a halogen lamp, white light laser has a wide flat continuum spectrum, which effectively reduces the diagnostic error of the SPR dip. In addition, the white light laser has a higher power per wavelength, which can improve the signal to noise ratio (SNR) of the SPR sensor.

**Figure 3:**
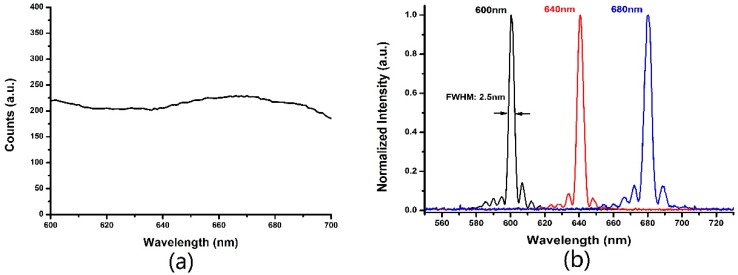
(**a**) The spectrum of white light laser at 600–700 nm; (**b**) The monochromatic light spectra filtered by AOTF. The center wavelengths are 600 nm/640 nm/680 nm.

In our experiments, the spectral band of 600–700 nm are filtered as excitation light; the corresponding spectral profile is shown in Figure 3. The stability of output power is less than 1%. AOTF is a kind of flexible, fast filter based on the effect of acousto-optics. It allows transmission of a very narrow band of light in a designated spectral range, and the center wavelength of the transmitted light can be fast tuned. In our experiment, an AOTF (AOTFnC-400.650-TN, AA Opto-electronic, Orsay, France) works in the range 300 to1000 nm with the variable bandwidth from 2 nm to 8 nm. To accurately measure bandwidth in the 600 nm to 700 nm range, we obtained a series of output spectral profiles at different wavelengths using a spectrometer. Several output spectral profiles with the FWHMs of 2.5 nm/4.2 nm/5.0 nm at the center wavelengths of 600 nm/640 nm/680 nm, respectively, are shown in Figure 3. Each wavelength’s monochromatic light power was about 0.3 mw. 

Light from the white light laser was coupled to the inverted prism through a multimodal optical fiber for SPR excitation. The AOTF fast scanned the incident wavelength step by step. In our experiments, we chose a wavelength step of 1 nm as this was found to have the lowest level of noise in the final detected signal [31]; along with this selection, the Kretschmann configuration was adopted: The SPR cell consisted of an equilateral prism made of BK7 glass, a microscope glass slide coated with 48 nm thick gold film, and a flow chamber for sample injection. The incident angle was manually set to an optimized value as calculated using Fresnel equations. In the present case, the angle for pure water with an excited wavelength of 660 nm is 72.5°. The output light is captured by a 12-bit CCD camera. It works in the spectral band of 380–950 nm and has 640 × 480 pixels with a frame rate of up to 125 fps.

The feedback loop shown in Figure 2 is designed to achieve automatic tracking of the shift in the SPR dip caused by binding of target biomolecules. Importantly, incorporation of this feedback loop drastically reduces the spectral width needed to sweep before hitting an SPR dip. Consequently, the amount of time required to arrive at an SPR resonance data point is significantly shortened. Typically, such spectral SPR systems continuously sweep through a fixed wavelength range. This ensure that the SPR dip never moves out of the range of interest in spite of a large change of refractive index due to a long interaction time, high analyte concentration levels or the large size of the target molecules. 

The response time for each wavelength of AOTF is about 1ms including control software running time; the framing rate is set to 90 fps to match the exposure time. In the process of the experiment, if the scanning spectral range is too large, the time of scanning will be increased; if the range is too small, the real resonance wavelength may not be obtained because of too few data points. After repeatedly testing, we set the x of the tracking algorithm to 15 nm in the work reported here, giving a scanning range at [*λ_i_* − 15 nm, *λ_i_* + 15 nm]. Each scan cycle was about 0.35 s. Each data point in our experiments is obtained by averaging the intensity values of 30 × 20 pixels for all SPR plots or 3 × 2 pixels for SPR images to reduce the effect of noise. The data analysis procedures were performed in an automatic mode using lab-built software based on numerical comparison techniques; this software included a filter algorithm to reduce the speckle noise in the system. 

## 3. Results and Discussion

### 3.1. Linearity and Sensitivity of SPR Measurement

**Figure 4:**
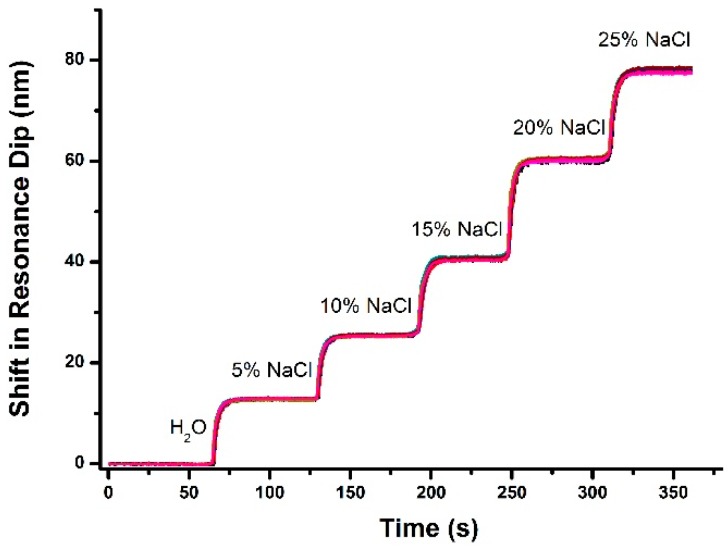
Shift of SPR dip versus salt concentration in water as detected by sensor sites within the 3 × 3 array.

To estimate the performance of our SPRi system, we measured the refractive index values of different salt-water mixtures. Salt solutions with concentration levels ranging from 0% to 25% in increments of 5% by volume (corresponding to a refractive index ranging from 1.3330 to 1.3793 RIU) were prepared [33]. On the sensing surface, the 3 × 3 array region was randomly selected. Figure 4 shows the step changes of resonant wavelength obtained from different salt concentrations and the corresponding shifts in spectral SPR dip. Based on measurement data obtained in the wavelength range from 600–700 nm, the measured dynamic range of the system is 4.63 × 10^−2^ RIU. The variation of each curve is basically the same, which shows that the sensor is consistent across its entire sensing surface.

To test the sensitivity of our SPRi system, we tested the low-concentration solution of sodium chloride. First, an incident angle was fixed and chosen as 72.5° in the prism based on the theoretical simulation of the SPR angular dip of pure water. Next, resonance wavelength changes were monitored in real time for 0.01%, 0.02%, and 0.05% aqueous solutions of sodium chloride.

In addition, the noise of system is obtained by measuring the shift of resonance using pure water as the sample. The root mean square noise of system is calculated based on the ten measurements. Three measurement results were brought into Equation (4) to calculate the average:

(4) $$\sigma_{RI}=\frac{\delta n}{\delta \lambda }\cdot \sigma_{SD}$$

where *σ_RI_* is the sensitivity limit, *δn* is the refractive index change of a bulk medium, *δλ* is the calculated wavelength shift, and *σ_SD_* is the root mean square noise of SPR system equal to an average of the ten resonance wavelength shifts obtained by measuring pure water. The calculated sensitivity of the system was 1.27 × 10^−6^ RIU.

### 3.2. Application to IgG Interaction Monitoring

To assess the capability of our SPRi system for monitoring biomolecular interactions, we performed real-time monitoring of binding interactions between goat anti-rabbit IgG and rabbit IgG. The sensor chip (15 mm × 15 mm) was washed with deionized water and dried by nitrogen. Sodium acetate was added to 5 mg/mL rabbit IgG to dilute it to 100 µg·mL^−1^.

Rabbit IgG solution was placed on the sensor chip surface and, after 30 min, formed an immobilized 3 × 2 array on the surface by physical adsorption. The chip was attached to the coupling prism using a small drop of refractive index matching liquid. A set of six spectral SPR plots representing the binding interactions taking place on the 3 × 2 sensing spots was generated from the sensor chip. We manually tuned the incidence angle to achieve minimum intensity at the SPR dip for a PBS sample.

**Figure 5:**
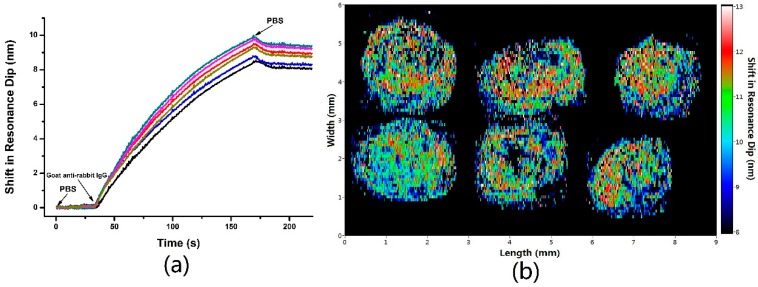
Measurement of antigen-antibody interaction between goat anti-rabbit IgG and rabbit IgG. (**a**) Real-time wavelength response of antigen-antibody binding reaction; (**b**) Image of SPR dip shift.

Ethanolamine buffer (pH = 8.5) was injected for 5 min deactivation (the flow rate of the peristalsis pump is 0.2 mL/min), so that no nonspecific binding of rabbit IgG occurred on the sensing surface. PBS 1× buffer was injected in the flow cell through a micropump at a speed of 25 µL/min for 3 min to obtain a steady baseline. The initial small scanning range was set at [*λ*_0min_ − 15 nm, *λ*_0max_ + 15 nm], in which *λ*_0min_, *λ*_0max_ were the minimum wavelength and maximum wavelength among the resonant wavelengths extracted from all of the SPR spectral profiles obtained from the sensing regions. After obtaining a steady baseline for several minutes, the process of capturing an SPR image was automatically executed for 1 scan to obtain the baseline spectral SPR image, which was produced through extracting the spectral SPR plot of individual sensor sites. Then goat anti-rabbit IgG solution of 10 µg·mL^−1^ was pumped into the sensor chip for 3 min. The subsequent antigen-antibody binding interaction occurring in the detection surface was monitored in real-time, as shown in Figure 5. After completion of binding interaction measurement, PBS 1× solution was pumped into the flow chamber for 2 min to ensure adequate buffer washing. After obtaining a steady baseline for several minutes, the system started capturing SPR image again after interaction in the same manner as before. The image of SPR dip shift can be obtained by comparing the SPR images before and after interaction. The color of each pixel in the picture represents the difference in the resonance wavelength before and after the interaction. Images of other sensor sites clearly reveal the presence of binding interactions as the SPR dips shift continuously.

## 4. Conclusions

We have demonstrated a fast wavelength-scanning SPR imaging technique with no mechanical moving parts. White light laser provides a high brightness and stability excitation light source. AOTF with a narrow bandwidth is used to achieve fast and flexible incident wavelength scan. By incorporating white light laser excitation and AOTF scanning with a feedback loop algorithm, the measurement time of SPR dip has dramatically shortened to 0.35 s for 2D array detections. With such a rapid scan, the RI resolution and measured dynamic range of our SPRi sensor, 1.27 × 10^−6^ and 4.63 × 10^−2^ RIU, are achieved. The antigen-antibody interaction results show our SPRi biosensor can be a useful platform for real-time and high-throughput high resolution analysis of biomolecular interactions on 2D arrays.
